# Association between overweight and central interleukin‐6 in a nonclinical adult population

**DOI:** 10.1002/npr2.12488

**Published:** 2024-10-14

**Authors:** Takako Enokida, Kotaro Hattori, Chiori Maeda, Takahiro Tomizawa, Hiroshi Kunugi

**Affiliations:** ^1^ Department of Bioresources, Medical Genome Center National Center of Neurology and Psychiatry Tokyo Japan; ^2^ Department of Mental Disorder Research, National Institute of Neuroscience National Center of Neurology and Psychiatry Tokyo Japan; ^3^ Department of Psychiatry Teikyo University School of Medicine Tokyo Japan

**Keywords:** body mass index, cerebrospinal fluid, interleukine‐6, neuroinflammation, obesity

## Abstract

**Aim:**

Overweight is associated with low‐grade systemic inflammation. However, its effect on neuroinflammation remains unclear. We examined the possible association between overweight and neuroinflammation using cerebrospinal fluid (CSF) in a nonclinical adult population in Japan.

**Methods:**

CSF and plasma levels of interleukin‐1β (IL‐1β), interleukin‐6 (IL‐6), tumor necrosis factor‐α (TNF‐α), plasma levels of C‐reactive protein (CRP), and leptin were measured in nonclinical adult participants (35 males and 34 females) who had no current or past history of neuropsychiatric diseases. We performed partial correlation analyses with sex and age as covariates between the body mass index (BMI) and the inflammatory markers and compared them between overweight and nonoverweight participants.

**Results:**

The BMI significantly correlated with CSF levels of IL‐6 (rs = 0.32, *p* = 0.009), plasma levels of CRP (rs = 0.30, *p* = 0.016), IL‐1β (rs = 0.29, *p* = 0.019), IL‐6 (rs = 0.25, *p* = 0.042), TNF‐α (rs = 0.43, *p* < 0.001), and leptin (rs = 0.72, *p* < 0.001). Overweight participants (BMI ≧ 25) had significantly higher CSF levels of IL‐6 (*p* < 0.001), plasma levels of IL‐1β (*p* = 0.023), TNF‐α (*p* < 0.001), and leptin (*p* < 0.001) than the nonoverweight participants.

**Conclusion:**

Overweight is associated with central IL‐6, a marker for neuroinflammation, as well as systemic inflammation markers, even in a nonclinical population.

## Abstract 2

The association between overweight and neuroinflammation was examined by measuring cerebrospinal fluid (CSF) levels of inflammatory markers in a nonclinical adult population. The analysis revealed a significant positive correlation between the BMI and CSF IL‐6 levels, with overweight participants showing significantly higher CSF IL‐6 levels.

## Graphical abstract

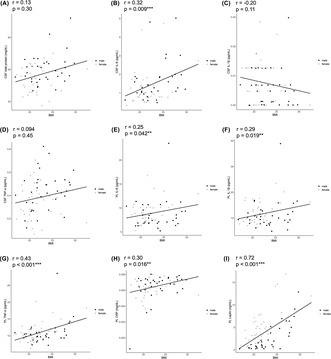

## 1 INTRODUCTION

Obesity causes low‐grade chronic systemic inflammation.^1^ Adipose tissue secretes inflammatory mediators such as interleukin‐6 (IL‐6), tumor necrosis factor‐α (TNF‐α), and C‐reactive protein (CRP), promoting infiltration of macrophages and other immune cells, leading to immune dysregulations. Recent research in clinical settings has suggested that inflammatory effects of obesity/overweight are not restricted to systemic but it also affects the central nervous system (CNS), and that obesity worsens neuroinflammation.^2^, ^3^, ^4^ However, correlation between obesity/overweight and neuroinflammation in nonclinical individuals remains unclear. In addition, to our knowledge, there is no study that examined the association between overweight and cerebrospinal fluid (CSF) proinflammatory cytokines in Asian population. Therefore, we measured inflammatory cytokine levels in CSF and plasma in nonclinical adult participants and examined the association between the body mass index (BMI) and overweight in order to determine whether obesity/overweight is also a risk factor for neuroinflammation.

## 2 METHODS

### 2.1 Participants and clinical assessments

Participants were recruited at the National Center of Neurology and Psychiatry (NCNP) Hospital, Tokyo, Japan, through website announcements. All participants were East Asians (most of them were Japanese) living in the Tokyo metropolitan area. A self‐reported medical history was taken to screen for complications, including heart, liver, and kidney diseases. Participants with a history of CNS disease or severe head injury were excluded. The participants underwent the Mini‐International Neuropsychiatric Interview (M.I.N.I.) to screen for psychiatric disorders.^5^ Both CSF and plasma samples were collected from the NCNP biobank as described previously.^6^ This study was conducted in accordance with the Declaration of Helsinki^7^ and was approved by the ethics committee of NCNP, Japan (A2012‐091 for biobanking and A2019‐092 for CSF collection and biomarker analyses). Written informed consent was obtained from every participant.

### 2.2 Laboratory examination of CSF

All CSF samples were screened using the general laboratory test for cell count, sugar, and total protein (TP), and samples within the normal range were used for analysis. A sample with an abnormally high cell count (> 500/μL) was excluded from the analysis.

### 2.3 Multiplex immunoassays

CSF IL‐1β, IL‐6, and TNF‐α concentrations were measured at Eurofins GeneticLab Co., Ltd. (Sapporo, Japan) using Bio‐Plex Pro™ Human Inflammation Panel 1, 3‐plex (Bio‐Rad Laboratories, Inc., Hercules, CA), and Luminex® 100/200™ System (Luminex Corp. Austin, TX). The CSF samples were diluted 1:2 and measured individually. Between‐plate normalization was performed using bridging samples (*n* = 16) from each plate. Plasma IL‐1β, IL‐6, TNF‐α, and CRP concentrations were measured at Acel, Inc. (Tokyo, Japan), using a Human Premixed Multi‐Analyte Kit (R&D Systems, Inc. MN) according to the manufacturer's instructions on a Bio‐Plex 200 system (Bio‐Rad Laboratories, Inc.) with low‐photomultiplier tube settings. Analyte concentrations were calculated using the Bio‐Plex Manager software (version 6.2.0.175, Bio‐Rad Laboratories, Inc.).

### 2.4 Statistical analysis

Partial correlation analysis with age and sex as covariates was performed between the BMI and inflammatory markers. Unpaired *t*‐test was used to compare the age and BMI between the overweight (BMI ≧ 25) and nonoverweight (BMI < 25) groups. Fisher's exact test was used to compare sex differences between the groups. Mann–Whitney *U*‐test was used to compare nonparametric comparisons between the groups. Statistical significance was set at *p* < 0.05. All analyses were conducted using SPSS (version 29, IBM Inc., Armonk, NY, USA).

## 3 RESULTS

**FIGURE 1:**
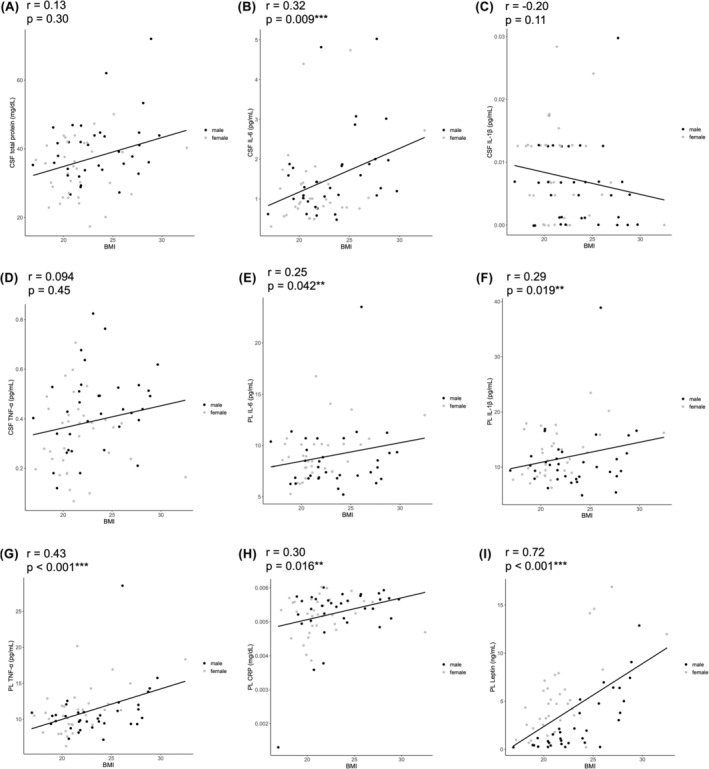
Scatter plots of inflammatory marker levels and body mass index (BMI). (A–I): Black and gray dots represent males and females, respectively. Straight lines represent regression lines. (A) Cerebrospinal fluid (CSF) total protein, (B) CSF IL‐6, (C) CSF IL‐1β, (d) CSF TNF‐α, (E) PL IL‐6, (F) PL IL‐1β, (G) PL TNF‐α, (H) PL CRP, and (I) PL leptin levels. **p < 0.05, ***p < 0.001.

Demographic data of the participants are shown in Table 1. Figure 1 shows scatter plots between the BMI and inflammatory markers. The BMI is not significantly correlated with CSF total protein levels but was significantly correlated with CSF IL‐6 levels (rs = 0.32, *p* = 0.009) by the partial correlation analysis. The BMI was not significantly correlated with CSF levels of IL‐1β or TNF‐α. The BMI correlated with plasma levels of CRP (rs = 0.30, *p* = 0.016), IL‐1β (rs = 0.29, *p* = 0.019), IL‐6 (rs = 0.25, *p* = 0.042), TNF‐α (rs = 0.43, *p* < 0.001), and leptin (rs = 0.72, *p* < 0.001) (Figure 1A–I). CSF IL‐6 levels were not correlated with any of the plasma inflammatory markers, nor did other CSF cytokines. None of the participants had a history of heart, liver, or kidney disease.

**TABLE 1:** Demographic data and levels of inflammatory markers of the participants.

Diagnosis (n)	Controls (68)	Overweight (15)	Nonoverweight (53)	*p*‐value
Age	43.1 ± 12.9	47.5 ± 13.5	41.9 ± 12.6	0.14
Sex (male%)	34 (50.0%)	11 (73.3%)	23 (43.4%)	0.077*
BMI	22.5 ± 3.3	27.6 ± 1.9	21.1 ± 1.8	<0.001***
CSF IL‐1β	0.0068 (0.0012–0.013)	0.0048 (0–0.0068)	0.0068 (0.0012–0.013)	0.23
CSF IL‐6	1.1 (0.8–1.8)	2.0 (1.2–3.0)	0.98 (0.75–1.42)	<0.001***
CSF TNF‐α	0.35 (0.26–0.45)	0.36 (0.35–0.45)	0.35 (0.26–0.45)	0.48
CSF TP	36 (30–42)	39.0 (36.0–45.0)	35.0 (0.26–0.45)	0.090*
Plasma IL‐1β	10.4 (8.3–13.7)	15.9 (9.3–16.4)	10.4 (8.3–12.9)	0.023**
Plasma IL‐6	8.5 (7.1–10.1)	9.3 (7.4–11.3)	7.9 (6.8–10.1)	0.065*
Plasma TNF‐α	10.5 (9.3–12.0)	12.4 (11.1–15.7)	10.1 (9.0–11.1)	<0.001***
Plasma CRP	0.0053 (0.0050–0.0057)	0.0056 (0.0052–0.0058)	0.0053 (0.0049–0.0056)	0.077*
Leptin	3095 (925–5993)	6954 (4759–11 973)	2121 (760–4727)	<0.001***

*Note*: For the age and BMI (body mass index), mean and standard deviation values are shown. For molecular levels of inflammatory markers, median values and inter‐quartile ranges are shown.

* *p* < 0.10.

** *p* < 0.05.

*** *p* < 0.01.

**FIGURE 2:**
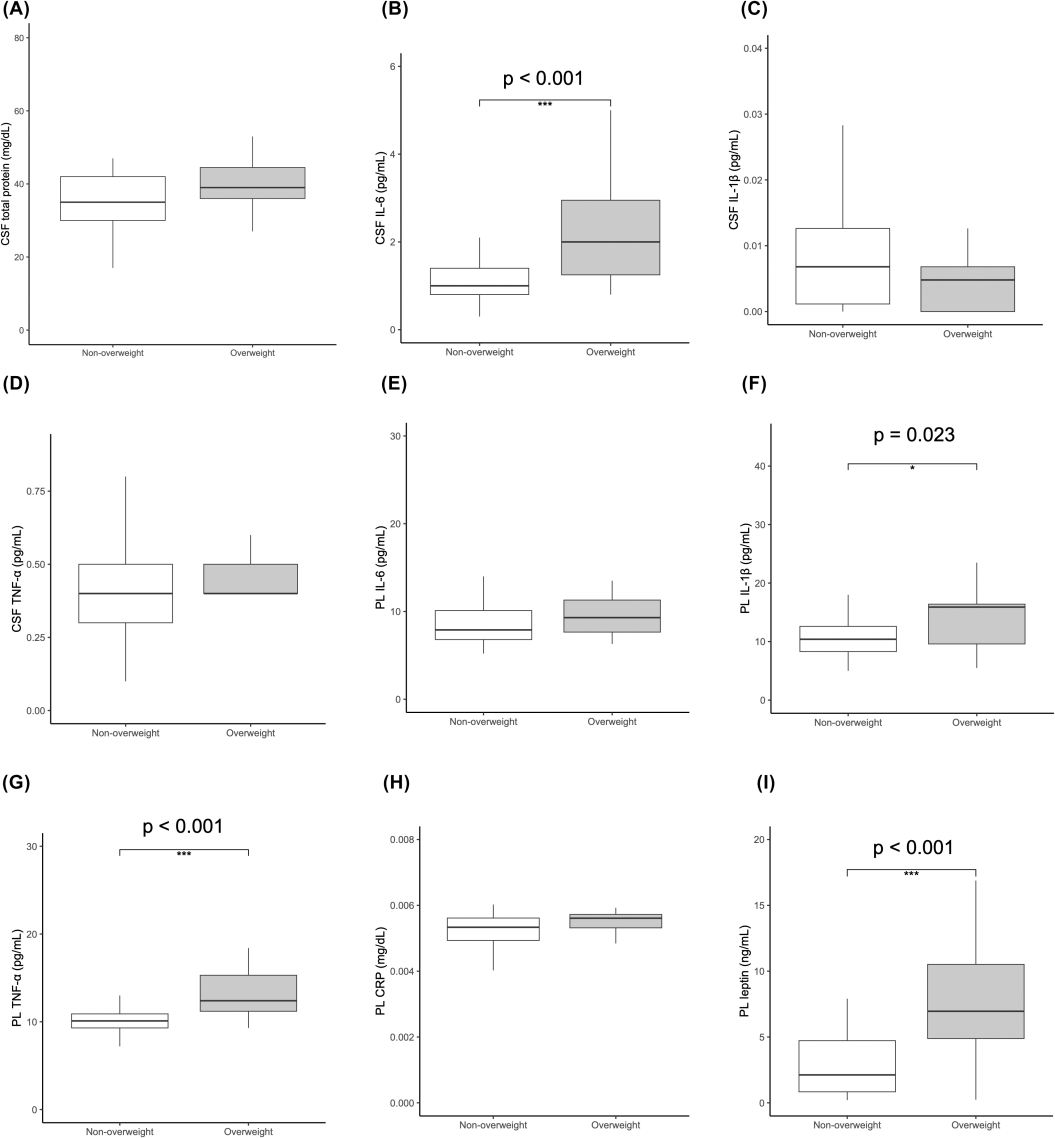
Box‐and‐whisker chart of inflammatory marker levels by the BMI. (A–I): BMI ≧ 25 as overweight and the BMI < 25 as nonoverweight. (A) CSF total protein, (B) CSF IL‐6, (C) CSF IL‐1β, (D) CSF TNF‐α, (E) PL IL‐6, (F) PL IL‐1β, (G) PL TNF‐α, (H) PL CRP, and (I) PL leptin levels. Medians and interquartile ranges are shown. *p < 0.01, ***p < 0.001.

Then we dichotomized the participants, i.e., overweight (BMI ≧ 25) and nonoverweight (BMI < 25), and compared the molecular levels between the two groups. Consistent with the results of the correlational analyses, the overweight group had significantly higher CSF levels of IL‐6 (*p* < 0.001), plasma levels of IL‐1β (*p* = 0.023), TNF‐α (*p* < 0.001) and leptin (*p* < 0.001) (Figure 2A–I).

## 4 DISCUSSION

The present study showed that the BMI positively correlated with CSF IL‐6 in nonclinical participants, and the overweight participants showed higher CSF IL‐6 levels than the nonoverweight ones, suggesting that obesity/overweight is associated with central inflammation. This is the first study that examined the association between overweight and CSF cytokine levels in a nonclinical population. Moreover, we obtained evidence for the first time in Asian people.

Overweight/obesity are known to be risk factors for both the development and severity of several neuropsychiatric diseases, such as depression,^8^ bipolar disorder,^9^ and Alzheimer's disease,^10^ and inflammation is considered to be an underlying mechanism. IL‐6 is a proinflammatory cytokine and it is produced by different cells in CNS, such as astrocyte, microglia, endothelial cells, and neurons.^11^ Elevated CSF IL‐6 is also reported in neuropsychiatric conditions.^12^, ^13^, ^14^, ^15^, ^16^ Our results suggested the possibility that overweight affects central inflammation, even in nonclinical people. A previous study on multiple sclerosis also reported that CSF IL‐6 and CSF leptin levels were higher in obese patients.^3^ Leptin, which is produced by adipocytes, is suggested to induce IL‐6 production.^17^ Leptin crosses into the CNS through the blood–brain barrier or choroid plexus, and plasma and CSF levels of leptin have been reported to be significantly correlated.^18^ As individuals get overweight, the plasma leptin levels may increase, leading to higher leptin levels that transfer to the CNS. These elevated CNS leptin levels may subsequently induce IL‐6 production in CNS, resulting in increased CSF IL‐6 levels secondary to overweight/obesity. IL‐6 is suggested to act in the hypothalamus and reduce appetite even under conditions of leptin resistance.^19^ Therefore, the increase in CSF IL‐6 may be a mechanism for maintaining homeostasis in response to overweight. CSF IL‐6 elevation has also been implicated in neuropsychiatric disorders, such as amyloid‐beta dynamics in Alzheimer's disorder.^20^ If overweight/obesity induces elevated CSF IL‐6 levels, this elevation may affect the brain and potentially increase the risk of neuropsychiatric disorders. However, previous studies reported a negative correlation between CSF IL‐6 and body fat mass in overweight patients with the BMI >29,^18^ and a decreasing body fat in obese rodents with intracerebroventricular IL‐6 treatment, which contradicts our findings. In the present study, there was no significant correlation between CSF IL‐6 and BMI in 15 participants with BMI > 29. One possible explanation of this discrepancy is that CSF IL‐6 levels increase to a certain BMI threshold, but may plateau or even decrease above the threshold. Overweight and obesity are affected by various factors, including ethnicity and dietary habits.^21^ Therefore, obesity /overweight may be heterogenous. For example, Asians have higher body fat percentage than Caucasians with equivalent BMI.^22^ The discrepancy with the previous study^18^ may be attributed in part to the heterogeneous nature of obesity/overweight which may have had diverse effects on CSF IL‐6 levels.

In the present study, the BMI was also positively correlated with plasma proinflammatory cytokines and leptin. This accords with previous reports that obesity is a potential risk factor for systemic inflammatory conditions.^23^, ^24^ Nevertheless, CSF IL‐6 levels were not correlated with any of the plasma inflammatory markers, nor did other CSF cytokines. This may suggest that elevated CSF IL‐6 associated with obesity/overweight is unrelated with peripheral IL‐6. Alternatively, there may be a discrepancy in the timing between peripheral and central inflammation. However, if overweight/obesity affects neuroinflammation, weight control interventions may prevent or reduce the risk of neuropsychiatric diseases in which neuroinflammation is thought to be involved. Further, incorporating weight control into treatment may also improve prognosis and symptoms.

Our study has several limitations. Since we did not perform general blood tests to screen for medical complications, the possibility remains that some obesity‐related complications, such as unmanifested fatty liver, may have influenced the results. Although we analyzed our data with sex as a covariate, females have generally higher body fat percentages than males and body fat percentage would be more accurate than BMI to detect the effect of being overweight. Another limitation was an insufficient statistical power because of the relatively small sample size. The lack of correlation of BMI with CSF IL‐1β or TNF‐α in this nonclinical population may result from a floor effect due to low baseline levels of these cytokines in the CSF. Memory impairment and anxiety‐like behavior mediated by TNF‐α in association with obesity was reported in mice^25^, ^26^; elevated TNF‐α might be associated with clinical symptoms, while elevated IL‐6 could represent an early stage of this process. Further research is needed to understand the association between obesity and neuroinflammation. Besides, participants in this study did not undergo psychological tests. Although the participants were nonclinical, it is possible that certain rating scales might detect differences in nonclinical behavior. However, we believe that our results may provide some insights into the association between obesity/overweight, peripheral inflammation, and neuroinflammation.

## 5 CONCLUSION

Overweight is positively associated with central IL‐6, a marker for neuroinflammation, as well as systemic inflammation markers, even in a nonclinical population.

## AUTHOR CONTRIBUTIONS

Conceptualization: K. H., and H. K.; formal analysis: T. E. and K. H.; investigation: T. E.; resources: T. E., K. H., C. M., and T. T.; writing—original draft preparation: T. E.; writing—review and editing: K. H.; supervision: K. H.; funding acquisition: K. H. All authors read and agreed to the published version of the manuscript.

## FUNDING INFORMATION

This study was supported by an Intramural Research Grant (3–1) for Neurological and Psychiatric Disorders of the NCNP from K.H., T.N., and K.N.

## CONFLICT OF INTEREST STATEMENT

The authors declare no conflict of interest.

## ETHICS STATEMENT

*Institutional Reviewer Board*: This study was approved by the ethics committee of NCNP, Japan (A2012‐091 for biobanking and A2019‐092 for CSF collection and biomarker analyses).

*Informed Consent*: Written informed consent was obtained from every participant.

*Registry and the Registration No. of the study/trial*: N/A.

## Supporting information


Table S1.

## Data Availability

The data that supports the findings of this study are available in the supplementary material of this article.

## References

[1] Ellulu MS , Patimah I , Khaza'ai H , Rahmat A , Abed Y . Obesity and inflammation: the linking mechanism and the complications. Arch Med Sci. 2017;13:851–863. 28721154 10.5114/aoms.2016.58928 PMC5507106

[2] Larsson A , Carlsson L , Lind A‐L , Gordh T , Bodolea C , Kamali‐Moghaddam M , et al. The body mass index (BMI) is significantly correlated with levels of cytokines and chemokines in cerebrospinal fluid. Cytokine. 2015;76:514–518. 26188367 10.1016/j.cyto.2015.07.010

[3] Stampanoni Bassi M , Iezzi E , Buttari F , Gilio L , Simonelli I , Carbone F , et al. Obesity worsens central inflammation and disability in multiple sclerosis. Mult Scler. 2020;26:1237–1246. 31161863 10.1177/1352458519853473

[4] Biechele G , Rauchmann B‐S , Janowitz D , Buerger K , Franzmeier N , Weidinger E , et al. Associations between sex, body mass index and the individual microglial response in Alzheimer's disease. J Neuroinflammation. 2024;21:30. 38263017 10.1186/s12974-024-03020-y PMC10804830

[5] Sheehan DV , Lecrubier Y , Sheehan KH , Amorim P , Janavs J , Weiller E , et al. The MINI‐international neuropsychiatric interview (MINI): the development and validation of a structured diagnostic psychiatric interview for DSM‐IV and ICD‐10. J Clin Psychiatry. 1998;59:22–33. 9881538

[6] Hattori K , Ota M , Sasayama D , Yoshida S , Matsumura R , Miyakawa T , et al. Increased cerebrospinal fluid fibrinogen in major depressive disorder. Sci Rep. 2015;5:11412. 26081315 10.1038/srep11412 PMC4469953

[7] World Medical Association . World medical association declaration of Helsinki: ethical principles for medical research involving human subjects. JAMA. 2013;310:2191–2194. 24141714 10.1001/jama.2013.281053

[8] Jokela M , Laakasuo M . Obesity as a causal risk factor for depression: systematic review and meta‐analysis of mendelian randomization studies and implications for population mental health. J Psychiatr Res. 2023;163:86–92. 37207436 10.1016/j.jpsychires.2023.05.034

[9] Goldstein BI , Liu S‐M , Zivkovic N , Schaffer A , Chien L‐C , Blanco C . The burden of obesity among adults with bipolar disorder in the United States: bipolar disorder and obesity. Bipolar Disord. 2011;13:387–395. 21843278 10.1111/j.1399-5618.2011.00932.x PMC3157038

[10] Ly M , Yu GZ , Mian A , Cramer A , Meysami S , Merrill DA , et al. Neuroinflammation: A modifiable pathway linking obesity, Alzheimer's disease, and depression. Am J Geriatr Psychiatry. 2023;31:853–866. 37365110 10.1016/j.jagp.2023.06.001 PMC10528955

[11] Erta M , Quintana A , Hidalgo J . Interleukin‐6, a major cytokine in the central nervous system. Int J Biol Sci. 2012;8:1254–1266. 23136554 10.7150/ijbs.4679 PMC3491449

[12] Hirohata S , Isshi K , Oguchi H , Ohse T , Haraoka H , Takeuchi A , et al. Cerebrospinal fluid interleukin‐6 in progressive neuro‐behçet's syndrome. Clin Immunol Immunopathol. 1997;82:12–17. 9000037 10.1006/clin.1996.4268

[13] Carpenter LL , Heninger GR , Malison RT , Tyrka AR , Price LH . Cerebrospinal fluid interleukin (IL)‐6 in unipolar major depression. J Affect Disord. 2004;79:285–289. 15023509 10.1016/S0165-0327(02)00460-3

[14] Lindqvist D , Janelidze S , Hagell P , Erhardt S , Samuelsson M , Minthon L , et al. Interleukin‐6 is elevated in the cerebrospinal fluid of suicide attempters and related to symptom severity. Biol Psychiatry. 2009;66:287–292. 19268915 10.1016/j.biopsych.2009.01.030

[15] Sasayama D , Hattori K , Wakabayashi C , Teraishi T , Hori H , Ota M , et al. Increased cerebrospinal fluid interleukin‐6 levels in patients with schizophrenia and those with major depressive disorder. J Psychiatr Res. 2013;47:401–406. 23290488 10.1016/j.jpsychires.2012.12.001

[16] Stampanoni Bassi M , Iezzi E , Landi D , Monteleone F , Gilio L , Simonelli I , et al. Delayed treatment of MS is associated with high CSF levels of IL‐6 and IL‐8 and worse future disease course. J Neurol. 2018;265:2540–2547. 30167879 10.1007/s00415-018-8994-5

[17] Tang C‐H , Lu D‐Y , Yang R‐S , Tsai H‐Y , Kao M‐C , Fu W‐M , et al. Leptin‐induced IL‐6 production is mediated by leptin receptor, insulin receptor substrate‐1, phosphatidylinositol 3‐kinase, Akt, NF‐kappaB, and p300 pathway in microglia. J Immunol. 2007;179:1292–1302. 17617622 10.4049/jimmunol.179.2.1292

[18] Stenlöf K , Wernstedt I , Fjällman T , Wallenius V , Wallenius K , Jansson J‐O . Interleukin‐6 levels in the central nervous system are negatively correlated with fat mass in overweight/obese subjects. J Clin Endocrinol Metab. 2003;88:4379–4383. 12970313 10.1210/jc.2002-021733

[19] Timper K , Denson JL , Steculorum SM , Heilinger C , Engström‐Ruud L , Wunderlich CM , et al. IL‐6 improves energy and glucose homeostasis in obesity via enhanced central IL‐6 trans‐signaling. Cell Rep. 2017;19:267–280. 28402851 10.1016/j.celrep.2017.03.043

[20] Lin H , Dixon SG , Hu W , Hamlett ED , Jin J , Ergul A , et al. p38 MAPK is a major regulator of amyloid Beta‐induced IL‐6 expression in human microglia. Mol Neurobiol. 2022;59:5284–5298. 35697992 10.1007/s12035-022-02909-0 PMC9398979

[21] Buckland G , Bach A , Serra‐Majem L . Obesity and the Mediterranean diet: a systematic review of observational and intervention studies. Obes Rev. 2008;9:582–593. 18547378 10.1111/j.1467-789X.2008.00503.x

[22] Chooi YC , Ding C , Magkos F . The epidemiology of obesity. Metabolism. 2019;92:6–10. 30253139 10.1016/j.metabol.2018.09.005

[23] Visser M , Bouter LM , McQuillan GM , Wener MH , Harris TB . Elevated C‐reactive protein levels in overweight and obese adults. JAMA. 1999;282:2131–2135. 10591334 10.1001/jama.282.22.2131

[24] Festa A , D'Agostino R Jr , Williams K , Karter AJ , Mayer‐Davis EJ , Tracy RP , et al. The relation of body fat mass and distribution to markers of chronic inflammation. Int J Obes Relat Metab Disord. 2001;25:1407–1415. 11673759 10.1038/sj.ijo.0801792

[25] Fourrier C , Bosch‐Bouju C , Boursereau R , Sauvant J , Aubert A , Capuron L , et al. Brain tumor necrosis factor‐α mediates anxiety‐like behavior in a mouse model of severe obesity. Brain Behav Immun. 2019;77:25–36. 30508579 10.1016/j.bbi.2018.11.316

[26] Melo HM , da Silva GSS , Sant'ana MR , Teixeira CVL , Clarke J , Miya Coreixas VS , et al. Palmitate is increased in the cerebrospinal fluid of humans with obesity and induces memory impairment in mice via pro‐inflammatory TNF‐α. Cell Rep. 2020;30:2180–2194. 10.1016/j.celrep.2020.01.072 32075735

